# Cardiac power output accurately reflects external cardiac work over a wide range of inotropic states in pigs

**DOI:** 10.1186/s12872-019-1212-2

**Published:** 2019-10-15

**Authors:** Dawud Abawi, Alessandro Faragli, Michael Schwarzl, Martin Manninger, David Zweiker, Karl-Patrik Kresoja, Jochen Verderber, Birgit Zirngast, Heinrich Maechler, Paul Steendijk, Burkert Pieske, Heiner Post, Alessio Alogna

**Affiliations:** 1 Department of Internal Medicine and Cardiology, Charité – Universitätsmedizin Berlin, Campus Virchow-Klinikum, Augustenburgerplatz 1, 13353 Berlin, Germany; 2 Berlin Institute of Health (BIH), Berlin, Germany; 3 DZHK (German Centre for Cardiovascular Research), partner site Berlin, Berlin, Germany; 4 Department of General and Interventional Cardiology, University Heart Center Hamburg-Eppendorf Martinistr 52, 20246 Hamburg, Germany; 5 DZHK (German Centre for Cardiovascular Research), partner site Hamburg/Kiel/Lübeck, Hamburg, Germany; 6 Department of Internal Medicine, Division of Cardiology, Medical University of Graz , Auenbruggerplatz 15, 8036 Graz, Austria; 7 Department of Cardiology, Heart Center Leipzig at University of Leipzig, Leipzig, Germany; 8 Leipzig Heart Institute at Heart Center Leipzig, Leipzig, Germany; 9 Department of Cardiothoracic Surgery, Medical University of Graz Auenbruggerplatz 29, 8036 Graz, Graz, Austria; 10 Department of Cardiology, Leiden University Medical Center, PO 9600, 2300 RC Leiden, The Netherlands; 11 Department of Internal Medicine and Cardiology, German Heart Center Berlin, Berlin, Germany; 12 Department of Cardiology, Contilia Heart and Vessel Centre, St. Marien-Hospital Mülheim, 45468 Mülheim, Germany

**Keywords:** Acute heart failure, Cardiac power output, Cardiogenic shock, Ejection fraction, Stroke work

## Abstract

**Background:**

Cardiac power output (CPO), derived from the product of cardiac output and mean aortic pressure, is an important yet underexploited parameter for hemodynamic monitoring of critically ill patients in the intensive-care unit (ICU). The conductance catheter-derived pressure-volume loop area reflects left ventricular stroke work (LV SW). Dividing LV SW by time, a measure of LV SW min^− 1^ is obtained sharing the same unit as CPO (W). We aimed to validate CPO as a marker of LV SW min^− 1^ under various inotropic states.

**Methods:**

We retrospectively analysed data obtained from experimental studies of the hemodynamic impact of mild hypothermia and hyperthermia on acute heart failure. Fifty-nine anaesthetized and mechanically ventilated closed-chest Landrace pigs (68 ± 1 kg) were instrumented with Swan-Ganz and LV pressure-volume catheters. Data were obtained at body temperatures of 33.0 °C, 38.0 °C and 40.5 °C; before and after: resuscitation, myocardial infarction, endotoxemia, sevoflurane-induced myocardial depression and beta-adrenergic stimulation. We plotted LVSW min^− 1^ against CPO by linear regression analysis, as well as against the following classical indices of LV function and work: LV ejection fraction (LV EF), rate-pressure product (RPP), triple product (TP), LV maximum pressure (LVP_max_) and maximal rate of rise of LVP (LV dP/dt_max_).

**Results:**

CPO showed the best correlation with LV SW min^− 1^ (*r*^2^ = 0.89; *p* < 0.05) while LV EF did not correlate at all (*r*^2^ = 0.01; *p* = 0.259). Further parameters correlated moderately with LV SW min^− 1^ (LVP_max_ *r*^2^ = 0.47, RPP *r*^2^ = 0.67; and TP *r*^2^ = 0.54). LV dP/dt_max_ correlated worst with LV SW min^− 1^ (*r*^2^ = 0.28).

**Conclusion:**

CPO reflects external cardiac work over a wide range of inotropic states. These data further support the use of CPO to monitor inotropic interventions in the ICU.

## Background

Benefits of acquiring hemodynamic information by pulmonary artery catheterization (PAC) in conditions of critical illness are controversial. Following several neutral studies arguing against the survival benefit of hemodynamic monitoring by PAC in different populations of critically ill patients [1–9], the use of PAC is not recommended as a diagnostic routine anymore [10, 11]. One possible explanation for the neutral effects of PAC is the lack of clear hemodynamic goals to guide evidence-based therapies [4]. A promising parameter for hemodynamic monitoring of critically ill patients is cardiac power output (CPO), which is assessed as the product of cardiac output (CO) and mean aortic pressure (MAP), divided by a constant of 451 [12]. Being the product of flow and pressure, CPO describes the function of the heart as a mechanical pump [13], representing the rate of external work done by the left ventricle. In addition, CPO has been shown to be the strongest independent predictor of intrahospital mortality in patients with cardiogenic shock [12] and to strongly correlate with outcome in chronic heart failure patients [14]. CPO might therefore be a relevant yet underexplored way to describe a patient’s hemodynamic state in the intensive care unit (ICU) setting [12].

The most comprehensive way to describe ventricular performance is pressure-volume analysis. Over the last decades, it became the gold standard to quantify cardiac function in vivo by providing measures that are reasonably load-independent, such as the end-systolic and end-diastolic pressure-volume relationships [15–17]. Pressure-volume analysis not only offers load-independent information, but it allows to quantify ventricular energetics and describes the interaction between heart and vasculature [18–20]. The area of the pressure-volume loop reflects the external work of the heart, also called left ventricular stroke work (LV SW). When LV SW is divided by time, a measure of LV SW per minute is obtained that shares the same unit as CPO (Watts). The correlation between CPO, as measured by PAC and invasive arterial pressure, and LV SW min^− 1^ measured via the invasive gold-standard (conductance method) has never been tested so far. The aim of the study was therefore to validate CPO as a marker of the actual LV SW min^− 1^ over a wide range of inotropic states. We therefore conducted a retrospective data analysis of previous animal studies from our group, in which we investigated the hemodynamic impact of mild hypothermia (MH, 33.0 °C) and hyperthermia (HT, 40.5 °C) by pressure-volume analysis in several porcine models of acute heart failure representative for patients in the ICU [21–24]. LV SW min^− 1^ was further compared with clinically established indices to describe LV function and work, such as left ventricular ejection fraction (LV EF), left ventricular maximum pressure (LVP_max_), maximum rate of rise of left ventricular pressure (LV dP/dt_max_), rate-pressure product [RPP = heart rate (HR) x LVP_max_] and triple product (TP = RPP x LV dP/dt_max_).

## Methods

The experimental protocols were approved by the local bioethics committee of Vienna, Austria (Austrian Committee for Animal Trials, in German “Tierversuchskommission”, BMWF-66.010/0091-II/3b/2013, BMWF-66.010/0033-II/10b/2008, BMWF-66.010/0103-II/10b/2009, BMWF-66.010/0108-II/3b/2010), and conform to the “European Convention for the Protection of Vertebrate Animals used for Experimental and other Scientific Purposes” (Council of Europe No 123, Strasbourg 1985). Landrace pigs were ordered from Heinz Stelzl, Großklein, Austria, and were delivered to the Institute for Biomedical Research of the Medical University of Graz (Roseggerweg 48, 8036 Graz, Austria). The experiments were performed either at the Section for Surgical Research, Department of Experimental Surgery (Auenbruggerplatz 15, 8036 Graz, Austria) or at the abovementioned Institute for Biomedical Research of the Medical University of Graz.

### Experimental setup

The experimental setup has been described before [25]. Briefly, Landrace pigs (*n* = 59, 68 ± 1 kg) were fasted overnight with free access to water and sedated with 0.25–0.5 mg kg^− 1^ midazolam (Midazolam „ERWO “5 mg/ml- ampoules, ERWO Pharma GmbH, Brunn am Gebirge, Austria) and 10–20 mg kg^− 1^ ketamine (Ketasol 100 mg/ml, aniMedica GmbH, Senden-Bösensell, Germany). The animals were intubated and anaesthesia was continued with 1.0–2.0 Vol% sevoflurane (Sevorane®, Abbott GmbH, Vienna, Austria), 30–35 μg kg^− 1^ h^− 1^ fentanyl (Fentanyl-Janssen™, 0.1 mg ampoules, Janssen-Cilag Pharma, Vienna, Austria), 1–1.25 mg kg^− 1^ h^− 1^ midazolam, 3 mg kg^− 1^ h^− 1^ ketamine and 0.2 mg kg^− 1^ h^− 1^ pancuronium (Pancouronium bromide 2 mg/ml ampoules, Ratiopharm GmbH, Ulm, Germany). Pigs were ventilated (Julian, Draeger, Vienna, Austria) with an FiO_2_ (Fraction of inspired oxygen) of 0.5, an I: E-ratio of 1:1.5, the positive end-expiratory pressure was set at 5 mmHg and a tidal volume (VT) of 10 ml kg^− 1^. The respiratory rate was adjusted constantly to maintain an end-expiratory carbon dioxide partial pressure between 35 and 45 mmHg. Under fluoroscopic guidance, all animals were instrumented with a Swan-Ganz catheter (Edwards Lifesciences CCO connected to Vigilance I, Edwards Lifesciences, Irvine, CA, USA), and an LV conductance catheter (5F, 12 electrodes, 7 mm spacing, MPVS Ultra, Millar Instruments, Houston, Texas, USA). A 14-F sheath was introduced into the left femoral vein, and an intravascular cooling catheter connected to a cooling unit (Accutrol™ Catheter 14F and InnerCool RTx Endovascular System, Philips Healthcare, Vienna, Austria) was positioned with the tip at the level of the diaphragm in the inferior caval vein. The body core temperature was measured at the tip of the Swan-Ganz-catheter. After instrumentation, the animals were allowed to stabilize for 30–60 min.

### Experimental protocols

**Fig. 1:**
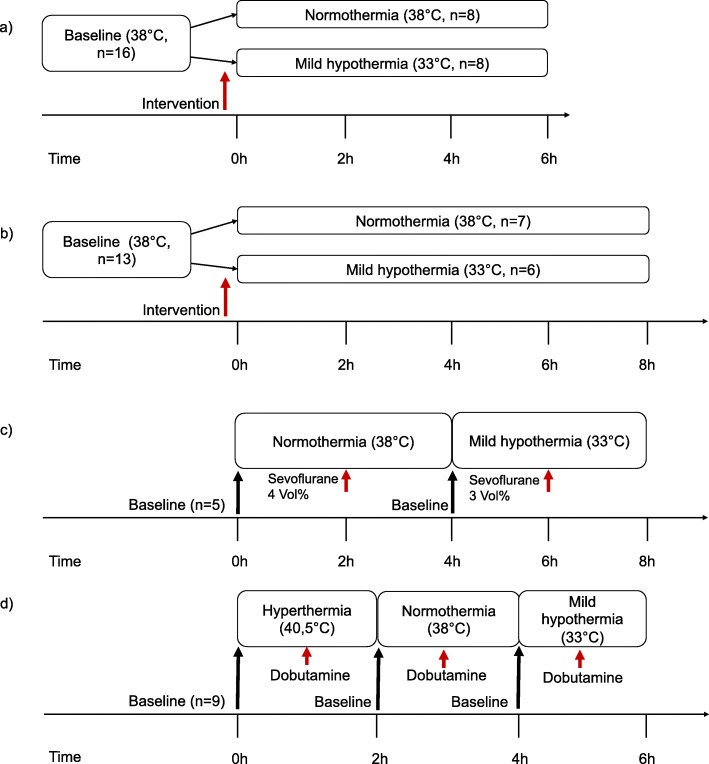
Experimental protocols: **a** Includes group 1–2 which are described as follows: group 1: resuscitation after ventricular fibrillation (RES, total *n* = 16), group 2: myocardial infarction by coronary microembolisation (CME, total *n* = 16); **b** group 3: endotoxemia by LPS-infusion (LPS, total *n* = 13); **c** group 4: sevoflurane-induced myocardial depression (SEVO, total *n* = 5); **d** group 5: Temperature modulation from hyperthermia to mild hypothermia vs dobutamine (DOB, total *n* = 9)

The experimental protocols are summarized in Fig. 1 and have been described in detail before [21–24].

Steady-state hemodynamics were obtained and averaged over three respiratory cycles. At each time point, volumetric conductance data were calibrated by measuring cardiac output (slope factor a) and hypertonic (10%) saline infusion (parallel volume) as described earlier [26, 27]. At the end of each experimental protocol anaesthetised and unconscious animals were euthanized by an injection of an 80 mmol potassium chloride bolus.

#### Group 1 – Resuscitation after ventricular fibrillation (RES)

Via a pacemaker lead, ventricular fibrillation (VF) was induced by applying an alternating current (50 Hz). After VF sustained for 5 min, resuscitation was begun by manual external chest compression at 80 min^− 1^, a single bolus of 15 mg kg^− 1^ adrenaline and biphasic defibrillation (200 J, Responder 2000; General Electric, Fairfield, CT, USA). Pigs (total *n* = 16) were assigned in a sequential 1:1 fashion to either normothermia (NT, *n* = 8, 38.0 °C) or MH (*n* = 8, 33.0 °C) as shown in Fig. 1 a. After 10 min past ROSC (return of spontaneous circulation), additional volume was given at either room temperature to normothermic animals or pre-cooled at 4 °C to cooled animals, consisting of 16 ml kg^− 1^ crystalloid solution and 8 ml kg^− 1^ hydroxyethyl starch (Voluven 6% 130/0.4; Fresenius, Austria) due to a substantial intravasal fluid loss. No further inotropes were given after ROSC [21]. For the following data analysis, the timepoints baseline and 6 h after return of spontaneous circulation were used.

#### Group 2 – Myocardial infarction after coronary microembolisation (CME)

Through repetitive slow (1 min) injections of 500,000 polystyrene microspheres (45 μm) into the proximal left circumflex coronary artery, myocardial infarction was induced. The injections were continued until cardiac power output was reduced by more than 40%. Three pigs developed sustained ventricular fibrillation during the injections and were excluded from the following analysis. The remaining animals (total *n* = 16) were assigned in a sequential 1:1 fashion to either NT (*n* = 8, 38.0 ^°^C) or MH (*n* = 8, 33.0 ^°^C) as shown in Fig. 1a [22]. We used baseline and 6 h after CME as timepoints for the data analysis.

#### Group 3 – Endotoxemia after LPS-infusion (LPS)

Following a Lipopolysaccharide (LPS)-infusion of 4 h, pigs (total *n* = 13) were monitored for another 4 h, resulting in a total of 8 h [23]. Assignment to either NT (*n* = 7, 38.0 °C) or MH (*n* = 6, 33.0 °C) in a sequential 1:1 fashion started with the beginning of LPS-infusion (Fig. 1b). With the onset of LPS-infusion cooling was started simultaneously in MH by the intravascular device and infusion of pre-cooled (4 °C) instead of a crystalloid solution at room temperature (10 ml kg^− 1^ h^− 1^ in both groups). When MAP fell below 55 mmHg during LPS-infusion, animals received up to four additional boluses of 500 ml crystalloid infusion, of which the last two were enriched with 1 μg kg^− 1^ epinephrine. When S_a_O_2_ (arterial oxygen saturation) fell below 90%, FiO_2_ was increased to 1.0 and the I: E ratio was changed to 1:1. When peak respiratory pressure reached 35 mmHg, endotracheal tube suction was performed, plus the tidal volume was reduced to 8 ml kg^− 1^ with a compensatory increase in respiration rate. For the following data analysis timepoints baseline and 8 h after onset of LPS infusion were used.

#### Group 4 – Sevoflurane-induced myocardial depression (SEVO)

After baseline measurements at NT (*n* = 5, 38.0 °C), the continuous administration of sevoflurane was elevated from a baseline of 1.0–2.0 to 4 Vol%. Subsequently, the continuous administration of sevoflurane was reduced to baseline values, and animals were cooled to MH (33.0 °C). After new baseline measurements in the same animals at MH, the continuous administration of sevoflurane was elevated from 1.0–2.0 to 3 Vol%. The Vol% of sevoflurane to induce cardiac depression during NT and MH was established in a series of pilot experiments. The sevoflurane dose was titrated aiming at an approximate 50% reduction of LV dP/dt_max_. A lower Vol% of sevoflurane was needed to induce the same magnitude of depression of contractile function during MH, probably due to different pharmacokinetics of the anesthetics at lower body temperatures, as previously reported [28–32]. The abovementioned timepoints (baseline at NT, 4 Vol% sevoflurane, baseline at MH and 3 Vol% sevoflurane) were considered for data analysis (Fig. 1c).

#### Group 5 – Temperature modulation from hyperthermia to mild hypothermia vs dobutamine (DOB)

A patient warming system (Bair Hugger, Warmtouch Series 500/OR, 3 M, Germany) with warming cover was used to increase and maintain the animals (total *n* = 9) body temperature at 40.5 °C (hyperthermia, HT). Only animals in this group were subsequently cooled from HT to NT and MH; at each temperature step baseline measurements were followed by a dobutamine-stress protocol until the steady state LV dP/dt_max_ was doubled (Fig. 1d) [24]. The dose of dobutamine necessary to double LV dP/dt_max_ decreased along with temperature from 2.1 ± 0.1 μg kg^− 1^ min^− 1^ (hyperthermia) to 1.8 ± 0.1 μg kg^− 1^ min^− 1^ (normothermia) and further to 1.5 ± 0.1 μg kg^− 1^ min^− 1^ (mild hypothermia). Baseline measurements at each temperature step and the corresponding measurements during dobutamine infusion were considered for data analysis.

### Data processing and statistical analyses

Details on data analysis have been described before [25]. Pressure-volume data and time intervals were analysed off-line by CircLab Software (custom made by P. Steendijk). End-diastole was defined as the time-point of zero crossing of LV dP/dt before its rapid upstroke. End-systole was defined as the time point of maximum pressure/volume ratio. CPO (W), LV SW min^− 1^ (W), LV EF, RPP and TP were calculated by using the following equations:

- CPO (W) = MAP x CO/451 [12]
- LV SW min^− 1^ (W) = [(LV SW x HR)/60] × 0.000133322
- LV EF (%) = Stroke volume (SV)/End-diastolic volume (EDV)
- RPP (mmHg min^− 1^) = HR x LVP_max_
- TP (mmHg^2^ min^− 1^ s^− 1^) = RPP x LV dP/dt_max_

All data are presented as mean ± standard deviation (SD). The correlations between LV SW and LV SW min^− 1^ with CPO, LV EF, RPP, TP, LVP_max_ and LV dP/dt_max_ were assessed by linear regression analysis. The assumption of normality of residuals was checked by the Kolmogorov-Smirnov test as well as graphically by a probability plot. Data between groups before and after cardiac insult at different temperatures were analysed by two-way ANOVA (groups 1–3). A one-way ANOVA for repeated measurements was used to compare data within one group (group 4). Steady state data at different temperatures, before and during dobutamine infusion, were compared by two-way ANOVA for repeated measurements (group 5). Post-hoc testing was performed by Tukey’s test. Normality was demonstrated by the Shapiro-Wilks test or by visual inspection of normal probability plots. Nonnormally distributed variables were examined either by Kruskal-Wallis- or Friedman-Test. A *p*-value < 0.05 was considered significant. For statistical calculations, we used the software Sigmastat (Version 4.0, Systat Software, Inc) and SPSS (Version 23.0, IBM, Armonk, NY).

## Results

### Systemic hemodynamics

Systemic hemodynamics from the experimental protocols are summarized in Table 1. CO, LV SW min^− 1^ and CPO spanned over a wide range of values, with the following means (ranges): CO 5.7 L min^− 1^ (8.2 L min^− 1^), CPO 0.94 W (1.75 W) and LV SW min^− 1^ 0.99 W (1.88 W).

**Table 1:** Systemic hemodynamics

Hemodynamic Parameter	Temp.	Timepoint	RES	CME	LPS	SEVO	Dob
HR	HT	Baseline					98 ± 12.8
(1 min^− 1^)		After Intervention					122 ± 10.1^c^
	NT	Baseline	89 ± 10.6	89 ± 11.8	97 ± 10	90 ± 14.7	89 ± 13.2
		After Intervention	84 ± 13.2	101 ± 18.5^*^	128 ± 17.4^*^	79 ± 9.6^*^	114 ± 10.2^a, c^
	MH	Baseline	86 ± 16.7	88 ± 10.8	98 ± 11.3	70 ± 10.1	65 ± 8^a, b^
		After Intervention	59 ± 10.7^*†^	67 ± 11.4^*†^	79 ± 11.6^*†^	60 ± 5.9^*^	83 ± 11.2^a, b, c^
MAP	HT	Baseline					74 ± 8.4
(mmHg)		After Intervention					77 ± 12.3
	NT	Baseline	92 ± 11.7	104 ± 17.3	86 ± 4.6	75 ± 7.1	68 ± 8
		After Intervention	64 ± 8.2^*^	54 ± 13^*^	53 ± 10.6^*^	44 ± 4.9^*^	71 ± 11.8
	MH	Baseline	90 ± 8.7	107 ± 10.3	85 ± 5.9	67 ± 7.7	65 ± 7.7^a^
		After Intervention	73 ± 15.4^*^	69 ± 6.7^*†^	58 ± 2.9^*^	42 ± 3.3^*^	69 ± 6.5^a^
CO	HT	Baseline					6.7 ± 0.9
(L min^−1^)		After Intervention					8.4 ± 1^c^
	NT	Baseline	5.7 ± 1.2	6.2 ± 0.9	6.8 ± 0.5	6.7 ± 0.6	6.1 ± 1
		After Intervention	4.8 ± 1.1	3.5 ± 0.7^*^	6.6 ± 1.3	5.0 ± 0,8^*^	7.8 ± 1.1^c^
	MH	Baseline	5.4 ± 1.3	6.5 ± 0.9	6.2 ± 0.8	4.9 ± 0.6	4.4 ± 0.6 ^*a, b*^
		After Intervention	3.5 ± 0.7^*†^	3.2 ± 0.5^*^	4.5 ± 1.22^*†^	4.4 ± 0.6	6.0 ± 0.85^a, b, c^
SVR	HT	Baseline					842 ± 152
(dyne.sec cm^−5^)		After Intervention					717 ± 170^c^
	NT	Baseline	1238 ± 401	1292 ± 345	931 ± 76	848 ± 97	856 ± 171
		After Intervention	915 ± 219	1089 ± 256	531 ± 83^*^	620 ± 117^*^	703 ± 128^c^
	MH	Baseline	1252 ± 310	1260 ± 153	1018 ± 139	1030 ± 137	1154 ± 202^a, b^
		After Intervention	1470 ± 384^*†*^	1618 ± 252^*†^	884 ± 206^*†*^	675 ± 183^*^	880 ± 88^a, b, c^
CPO	HT	Baseline					1.10 ± 0.2
(W)		After Intervention					1.40 ± 0.3^c^
	NT	Baseline	1.14 ± 0.2	1.43 ± 0.3	1.29 ± 0.1	1.12 ± 0.2	0.90 ± 0.2
		After Intervention	0.69 ± 0.24^*^	0.42 ± 0.2^*^	0.80 ± 0.3^*^	0.48 ± 0.1^*^	1.20 ± 0.3^a, c^
	MH	Baseline	1.09 ± 0.3	1.55 ± 0.3	1.17 ± 0.2	0.72 ± 0.1	0.60 ± 0.1^a, b^
		After Intervention	0.58 ± 0.2^*^	0.49 ± 0.1^*^	0.58 ± 0.2^*^	0.40 ± 0.0^*^	0.90 ± 0.2^a, b, c^

All values are presented as mean ± standard deviation (SD). *Temp.* Temperature, *HT* Hyperthermia, *NT* Normothermia, *MH* Mild hypothermia, *RES* Resuscitation after ventricular fibrillation, *CME* Coronary microembolisation, *LPS* Endotoxemia by Lipopolysaccharide (LPS)-infusion, *SEVO* Sevoflurane-induced myocardial depression, *Dob* Temperature modulation from hyperthermia to mild hypothermia vs. dobutamine (intervention) at each temperature step, *HR* Heart rate, *MAP* Mean aortic pressure, *CO* Cardiac Output, *SVR* Systemic vascular resistance, *CPO* Cardiac Power Output

*p < 0.05 vs baseline; ^†^*p* < 0.05 vs NT; ^a^*p* < 0.05 vs hyperthermia; ^b^*p* < 0.05 vs normothermia; ^c^*p* < 0.05 vs baseline at each temperature step

During mild hypothermia, heart rate decreased (*p* < 0.05) in all animals and was significantly lower at MH than during normothermia or hyperthermia after each intervention. Dobutamine infusion increased heart rate at each temperature step.

Mean aortic pressure decreased with all interventions and was more preserved during MH after myocardial infarction (*p* < 0.05).

In group 5 (DOB), CO and CPO decreased with cooling from hyperthermia to normothermia to MH and were increased by dobutamine at each temperature step (Table 1). In groups 1–4, CO was per se significantly lower during MH than during NT. CPO decreased significantly with the interventions in groups 1–4, but there was no significant difference between NT and MH.

Systemic vascular resistance increased with cooling from hyperthermia to MH and was decreased by dobutamine at each temperature step. Except for sevoflurane-induced myocardial depression, SVR was significantly higher during MH after each intervention.

### Cardiac power output and other parameters of cardiac function as indices of left ventricular stroke work

**Fig. 2:**
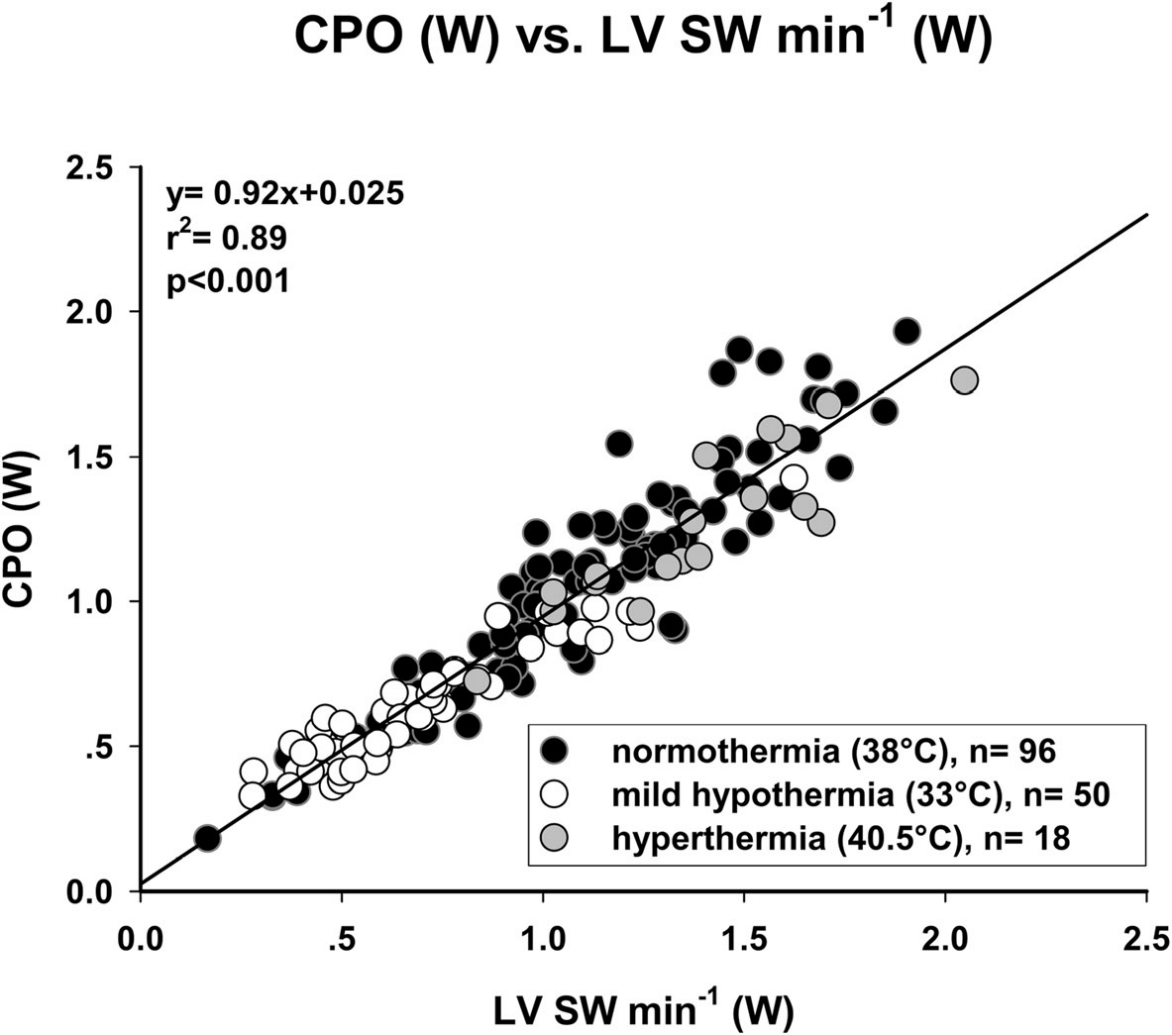
Cardiac Power Output (CPO) accurately reflects Left ventricular stroke work per minute (LV SW min^− 1^) over a wide range of inotropic states. Any rise or fall of LV SW min^− 1^ corresponds to an equivalent change of CPO

**Fig. 3:**
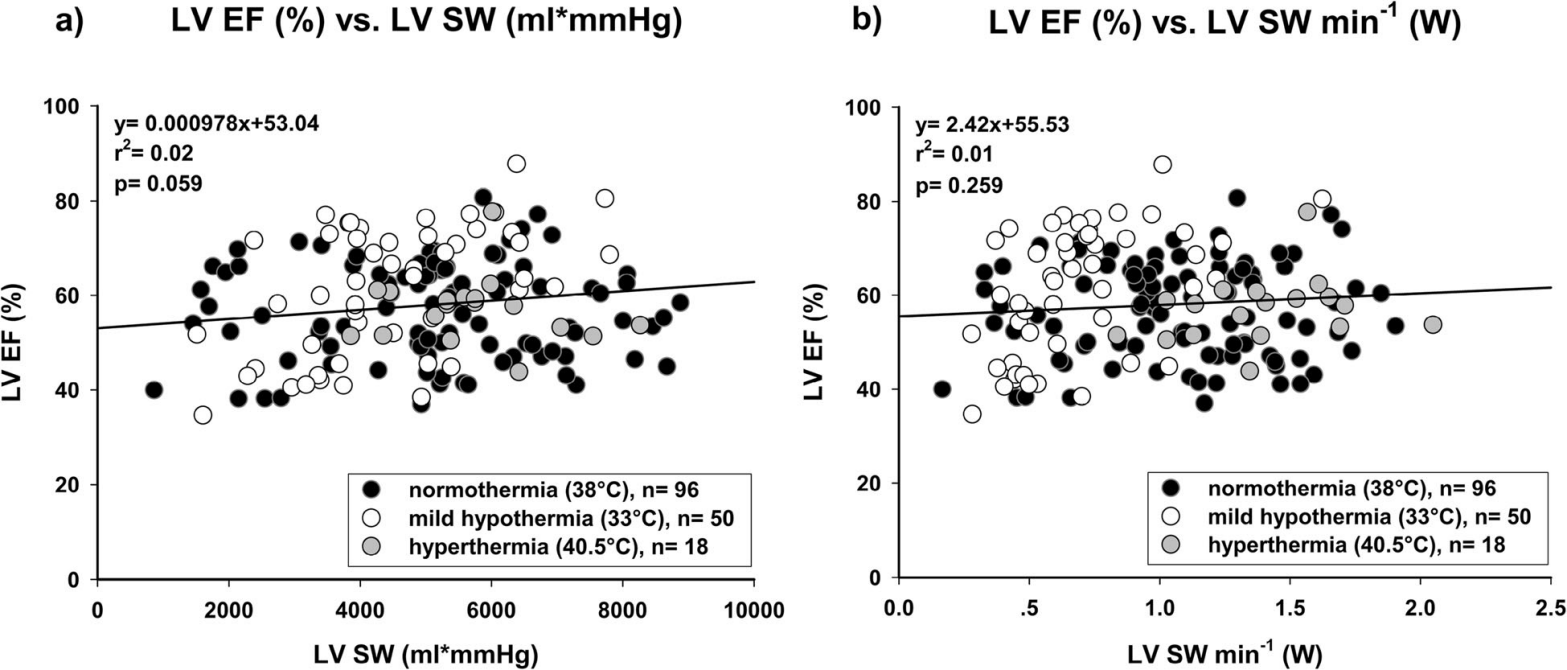
Left ventricular ejection fraction (LV EF) did not correlate with (**a**) Left ventricular stroke work per beat (LV SW) nor with (**b**) Left ventricular stroke work per minute (LV SW min^− 1^)

**Fig. 4:**
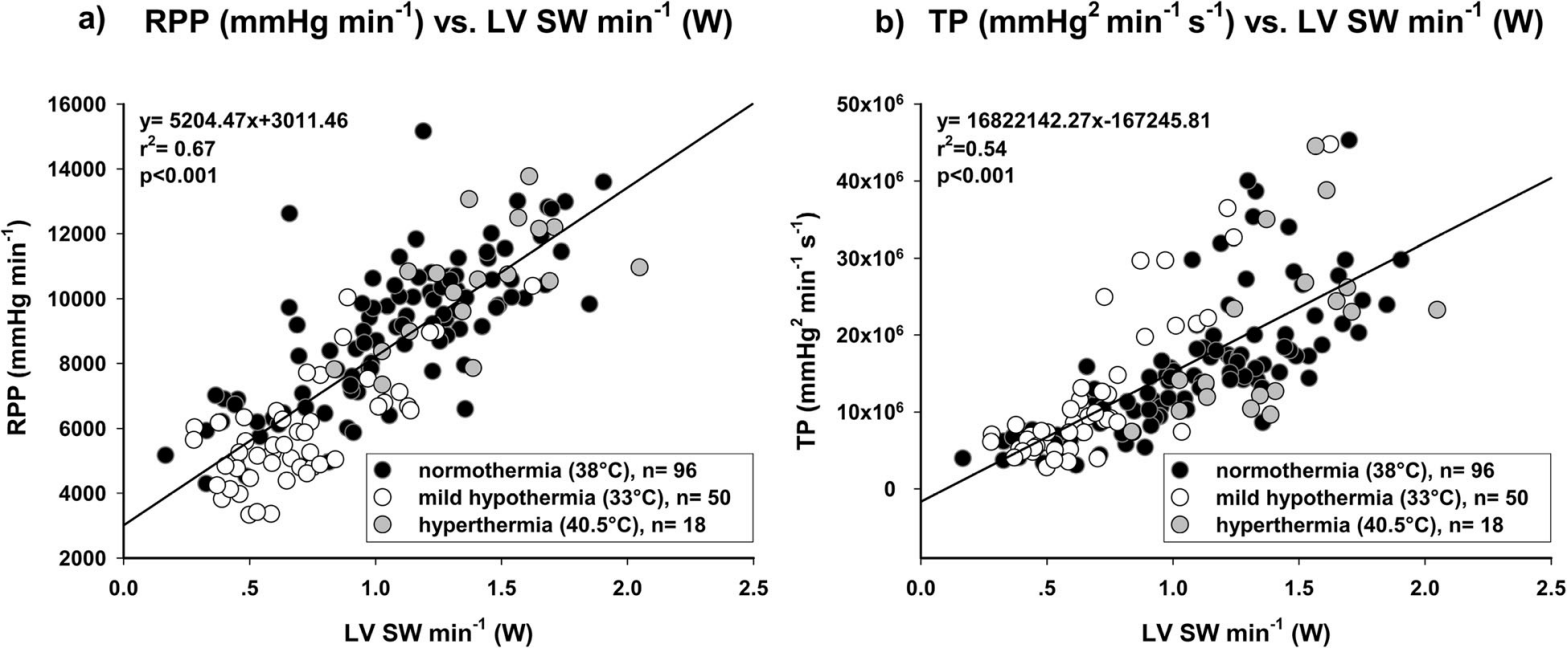
Both (**a**) Rate Pressure Product (RPP) and (**b**) Triple Product (TP) correlated moderately with Left ventricular stroke work per minute (LV SW min^− 1^)

**Fig. 5:**
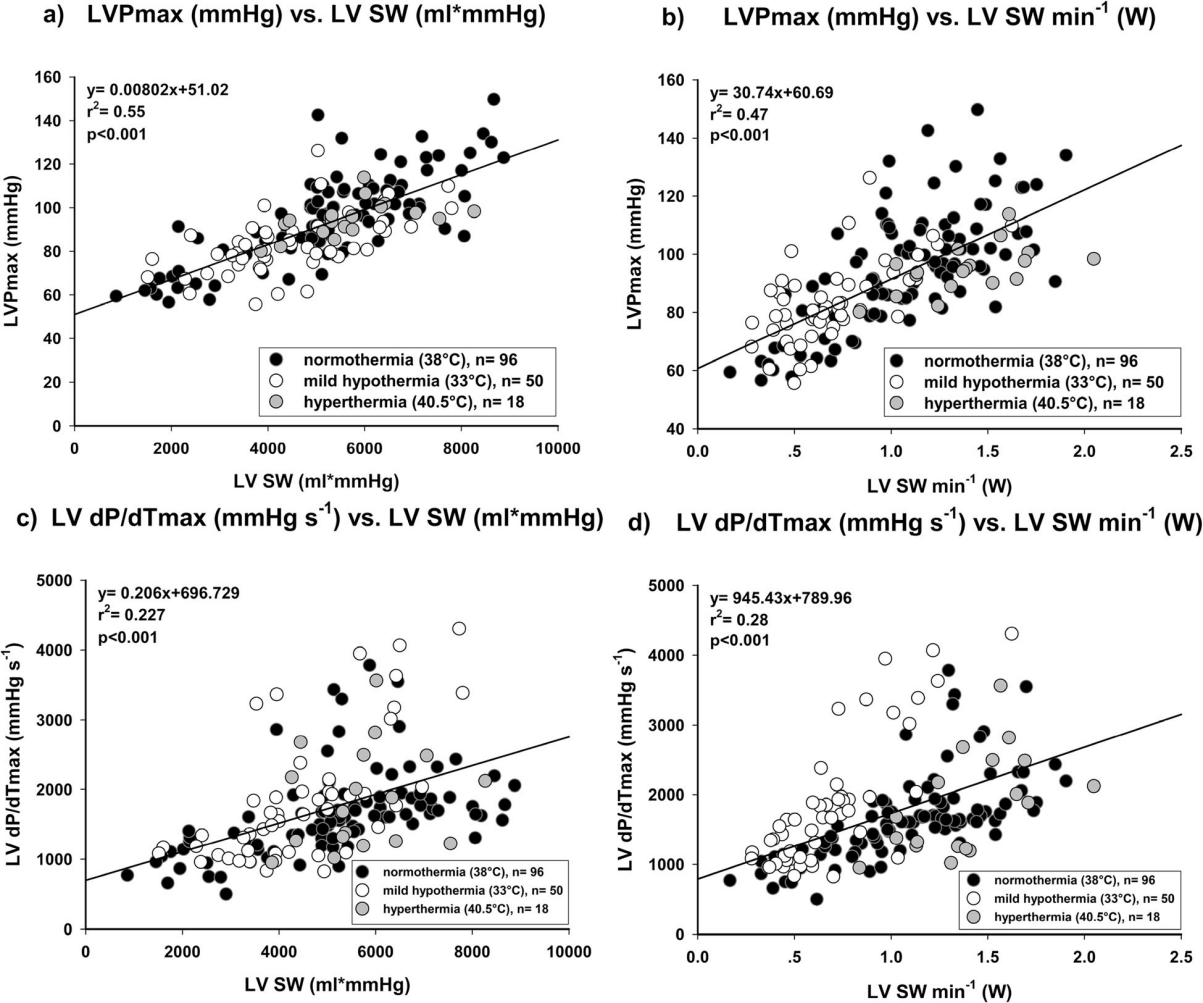
LV maximum pressure (LVP_max_) showed a better correlation with both (**a**) Left ventricular stroke work per beat and (**b**) Left ventricular stroke work per minute (LV SW min^− 1^) than maximum rate of rise of LVP (LV dP/dT_max_, **c** and **d**)

CPO showed the best correlation with LV SW min^− 1^ (Fig. 2; *r*^2^ = 0.89; *p* < 0.001). LV EF did not correlate at all, neither with LV SW (Fig. 3a; *r*^2^ = 0.02; *p* = 0.059) nor with LV SW min^− 1^ (Fig. 3b; *r*^2^ = 0.01; *p* = 0.259). Other common parameters of cardiac function showed a statistically significant but moderate correlation. RPP (Fig. 4a; *r*^2^ = 0.67; *p* < 0.001) did correlate better than TP (Fig. 4b; *r*^2^ = 0.54; *p* < 0.001) with LV SW min^− 1^. LVP_max_ showed a similar correlation with LV SW (Fig. 5a; *r*^2^ = 0.55; *p* < 0.001), but correlated worse with LV SW min^− 1^ (Fig. 5b; *r*^2^ = 0.47; *p* < 0.001). LV dP/dt_max_ showed the worst correlation with both LV SW (Fig. 5c; *r*^2^ = 0.23; *p* < 0.001) and LV SW min^− 1^ (Fig. 5d; *r*^2^ = 0.28; *p* < 0.001). An additional file shows the correlations between CPO and LV SW min^− 1^ of each individual experimental group (Additional file 1).

## Discussion

Here, we demonstrate that Cardiac Power Output accurately reflects left ventricular stroke work per minute over a wide range of inotropic states. Data were collected from experimental studies of the hemodynamic impact of mild hypothermia on acute heart failure models, such as 1) resuscitation after ventricular fibrillation, 2) coronary no-reflow infarction, 3) endotoxemia by LPS-infusion, 4) sevoflurane-induced myocardial depression and 5) beta-adrenergic stimulation via dobutamine infusion at different core-body temperatures. We therefore investigated conditions ranging from hypo- to hypercontractile LV function. CPO showed a high positive correlation over the whole range of inotropic states with LV SW min^− 1^, making it a valid parameter to monitor inotropic interventions in the ICU setting.

### Cardiac power output and left ventricular stroke work

The correlation between CPO, as measured by pulmonary artery catheterization (PAC) and invasive arterial pressure, and LV SW min^− 1^ measured via the invasive gold-standard (conductance method) has never been tested so far. Since pressure-volume analysis remains a time-consuming, complex and highly invasive measurement requiring additional equipment and expertise, the concept of deriving similar information by assessing CPO instead of LV SW is therefore quite attractive for the clinical practice. Being assessed as the product of cardiac output and mean aortic pressure and therefore combining the heart’s ability to create both pressure and flow, CPO describes the function of the heart as a hydraulic pump [13]. More precisely, hydraulic power in general consists of two components. The power expended to generate a steady (or non-pulsatile) flow is described as the mean external power, which is assessed as the product of mean arterial pressure and cardiac output. The energy used in producing the pulsatile component of flow and pressure is described as pulsatile power. The sum of both mean and pulsatile power results in the total external hydraulic power generated by the ventricle [33]. Hence, CPO is a measure of mean power, whereas LV SW is a measure of total energy imparted to the vasculature system including pulsatile power being assessed on a beat to beat basis. Under physiological conditions, the fraction of pulsatile power is about 10% of the total power generated by the left ventricle, while it rises with arterial hypertension, decreased distensibility of the proximal arteries and increases in heart rate [34–36]. In the pulmonary circulation, pulsatile power can make up to 30% of total power [33, 37]. The ratio between pulsatile power and mean power can be regarded as a measure of the efficiency of ventricular-arterial coupling, while an increase of the pulsatile fraction indicates a decrease in efficiency [35]. Although CPO does not include information of pulsatile power, we could show here for the first time a strong correlation to LV SW min^− 1^ under various inotropic states during acute heart failure. Of note, none of the experiments included LV output tract (LVOT) obstruction or aortic valve obstruction as well as severe stiffness of the aorta, which in turn would have increased LV SW min^− 1^ without changing CPO.

As abovementioned, CPO was shown to be the best predictor of intrahospital mortality in patients with cardiogenic shock [12]. Furthermore, it allows an exact hemodynamic characterization of patients with acute congestive heart failure by plotting CPO with SVR [38] and it was shown to be the only parameter, when measured at baseline, with a significant prediction of recurring severe acute heart failure (manifested as frank pulmonary edema) within 24 h from hospitalization in the ICU setting [39]. However, the role of CPO has not been yet fully integrated into the clinical ICU routine, in part due to a lack of data supporting the use of PAC in patients with advanced heart failure. In a meta-analysis investigating 13 randomized controlled trials (RCT) on the impact of PAC on survival, Shah et al. [4] argued that the neutral effects of PAC might be based on unclear hemodynamic goals in combination with a lack of effective treatment strategies driven by the obtained hemodynamic measurements [2, 4]. We suggest here that CPO obtained via PAC is an excellent parameter to monitor cardiac performance in experimental conditions resembling critically-ill patients. Furthermore, recent data show the feasibility of a minimally invasive assessment of CPO and cardiac power integral [40–43], opening new scenarios of CPO-based monitoring of critical patients without the need for PAC.

### Left ventricular ejection fraction

We could clearly show that LV EF does not reflect external cardiac work over a wide range of experimental inotropic states representative for patients during acute heart failure. In clinical practice, LV EF plays a major role in the diagnosis and treatment of heart failure, as a surrogate marker of cardiac remodelling [44] and reverse remodelling [45] or as a general predictor of outcome in heart failure with reduced LV EF [46, 47]. Nevertheless, our data imply that LV EF is not accurate enough in mirroring the function of the heart as a hydraulic pump under various conditions of acute heart failure and should be preferably interpreted within the hemodynamic context.

### LV pressure and pressure-derived indices of cardiac workload

The rate pressure product and the triple product are both clinical indices of myocardial oxygen consumption and therefore regarded as indirect measures of cardiac work [48–52]. Thus, we used these parameters as surrogates for LV SW min^− 1^. In our findings, we confirm a correlation between both RPP and TP with LV SW min^− 1^, with RPP having a better correlation than TP. The worse correlation of TP with LV SW min^− 1^ could be explained by integrating LV dP/dt_max_ into the calculation which is known to be a preload, afterload and heart rate dependent measure of cardiac contractility [44, 53–55]. Also, LV dP/dt_max_ itself correlated worst with LV SW and LV SW min^− 1^.

Interestingly, LVP_max_ alone correlated as good as the TP with LV SW, emphasizing the potential role of estimating LV pressure as a measure of the actual work done by the left ventricle in specific subpopulations of cardiogenic shock patients, i.e. under left ventricular assistance.

### Limitations

The main limitation is the retrospective design of the study, meaning that the analysed data were not always part of the major outcome of the original studies. However, the quality of the original tracings has been reviewed by 2 independent investigators. Specific limitations related to the experimental setup from which these data were derived have been described earlier [21–24]. Another limitation of the study is related to the fact that animals were investigated under general anaesthesia, in order to minimize the animals’ distress and to obtain stable hemodynamic conditions. Nevertheless, we believe the data to be representative enough for the translation to a clinical ICU setting. Furthermore, the study did not include experimental models of LVOT obstruction or increased stiffness of the aorta for modelling of patients with aortic stenosis or severe arteriosclerosis.

## Conclusions

CPO is an excellent parameter of external cardiac work over a wide range of inotropic states, while clinically established indices to describe cardiac function, such as LV ejection fraction, rate-pressure product and triple product perform poorly.

These data further support the use of CPO to monitor cardiac workload as well as the effect of inotropic interventions in critically ill patients in the ICU setting. Clinical studies will address the impact of CPO on improving patients’ survival.

## Supplementary information

**Additional file 1.** Individual correlations of CPO (W) vs. LVSW min^− 1^ (W). Cardiac Power Output (CPO) significantly reflects Left ventricular stroke work per minute (LV SW min-1) over a wide range of inotropic states in each experimental group (Group 1–5). Groups are described as follows: a) group 1: resuscitation after ventricular fibrillation (RES); b) group 2: myocardial infarction by coronary microembolisation (CME); c) group 3: endotoxemia by LPS-infusion (LPS); d) group 4: sevoflurane-induced myocardial depression (SEVO); e) group 5: Temperature modulation from hyperthermia to mild hypothermia vs dobutamine (DOB). Any rise or fall of LV SW min-1 corresponds to an equivalent change of CPO.

## Data Availability

The datasets used and/or analysed during the current study are available from the corresponding author on reasonable request.

## Abbreviations

CME: Coronary microembolisation
CO: Cardiac output
CPO: Cardiac power output
DOB: Temperature modulation from hyperthermia to mild hypothermia vs dobutamine
FiO_2_: Fraction of inspired oxygen
HT: Hyperthermia
I:E: Ratio of inspiration to expiration
ICU: Intensive-care unit
LPS: Lipopolysaccharide
LV dP/dt_max_: Maximal rate of rise of LVP
LV EF: LV ejection fraction
LV SW: Left ventricular stroke work
LVOT: LV output tract
LVP_max_: LV maximum pressure
MAP: Mean aortic pressure
MH: Mild hypothermia
NT: Normothermia
PAC: Pulmonary artery catheterization
RCT: Randomized controlled trial
RES: Resuscitation after ventricular fibrillation
ROSC: Return of spontaneous circulation
RPP: Rate-pressure product
S_a_O_2_: Arterial oxygen saturation
SEVO: Sevoflurane-induced myocardial depression
TP: Triple product
VF: Ventricular fibrillation
VT: Tidal volume
