# Supplementary figures and images for: Cardiac power output accurately reflects external cardiac work over a wide range of inotropic states in pigs
Source: BMC Cardiovasc Disord. 2019 Oct 15;19:217. doi: 10.1186/s12872-019-1212-2 (PMC6792198; doi:10.1186/s12872-019-1212-2)

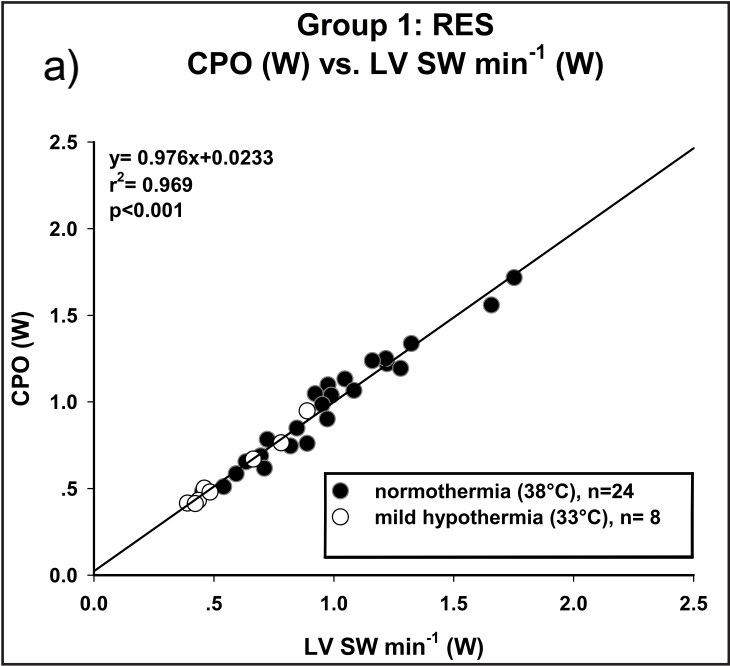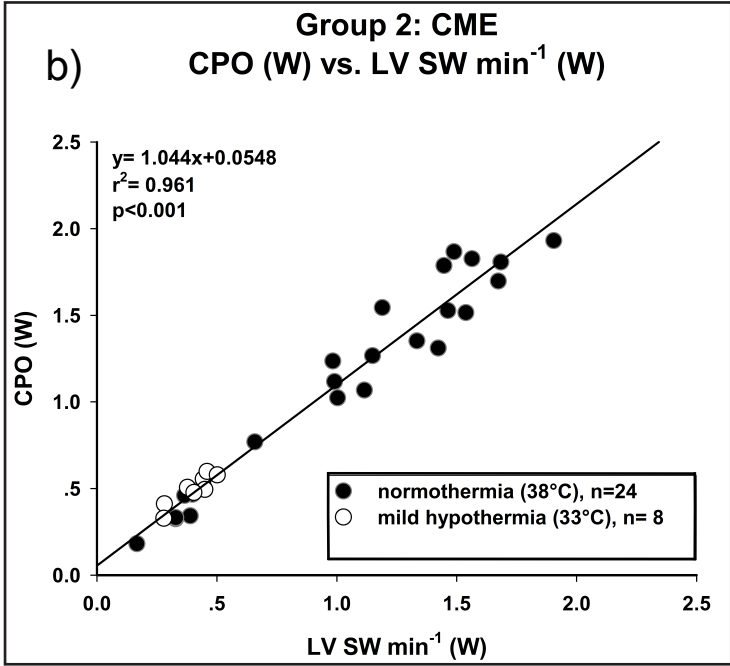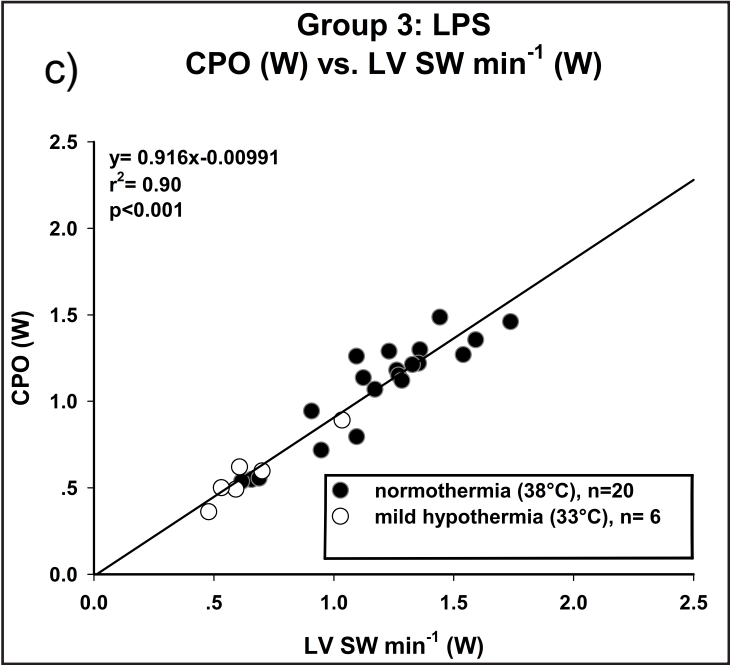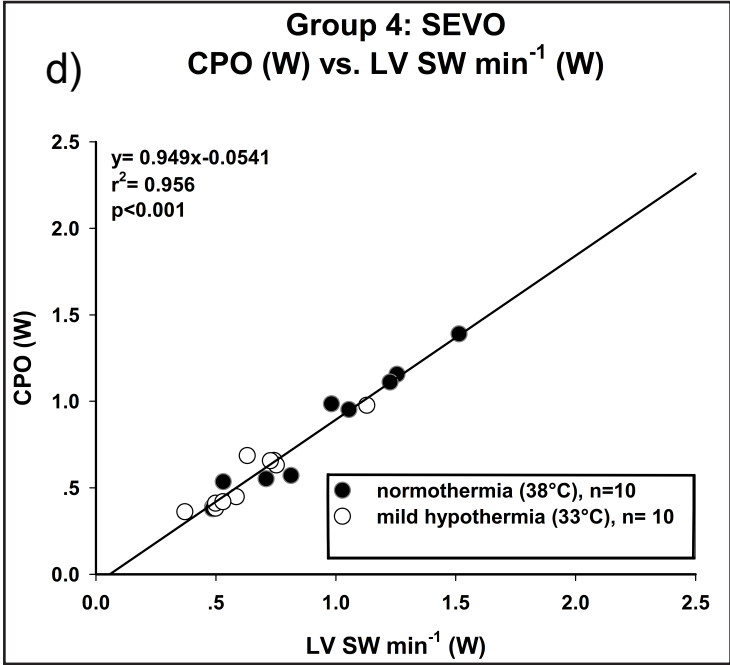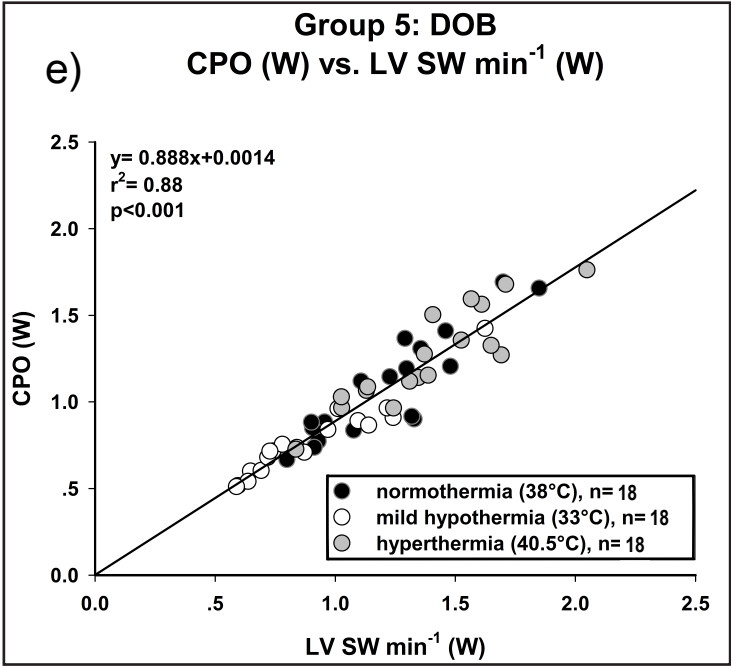

Supplement: Supplementary file 1 — Additional file 1. Individual correlations of CPO (W) vs. LVSW min− 1 (W). Cardiac Power Output (CPO) significantly reflects Left ventricular stroke work per minute (LV SW min-1) over a wide range of inotropic states in each experimental group (Group 1–5). Groups are described as follows: a) group 1: resuscitation after ventricular fibrillation (RES); b) group 2: myocardial infarction by coronary microembolisation (CME); c) group 3: endotoxemia by LPS-infusion (LPS); d) group 4: sevoflurane-induced myocardial depression (SEVO); e) group 5: Temperature modulation from hyperthermia to mild hypothermia vs dobutamine (DOB). Any rise or fall of LV SW min-1 corresponds to an equivalent change of CPO. [file 12872_2019_1212_MOESM1_ESM.pdf]
